# Risk factors for ICU admission in hospitalized children with respiratory syncytial virus infection

**DOI:** 10.3389/fcimb.2026.1834056

**Published:** 2026-05-21

**Authors:** Chunyun Fu, Fengkui Liang, Lishai Mo, Junming Lu, Lujin Tang, Jiangyang Zhao, Xuehua Hu, Yanmei Li, Yuchen Wei, Chengxiang Tang, Dejiu Nong, Jinqian Luo, Xiaoying Chen, Shaoling Wei

**Affiliations:** 1 Medical Science Laboratory, Children’s Hospital, Maternal and Child Health Hospital of Guangxi Zhuang Autonomous Region, Nanning, China; 2 Department of Intensive Care Medicine, Children’s Hospital, Maternal and Child Health Hospital of Guangxi Zhuang Autonomous Region, Nanning, China; 3 Respiratory Diagnosis and Treatment Center, Children’s Hospital, Maternal and Child Health Hospital of Guangxi Zhuang Autonomous Region, Nanning, China; 4 Department of Nephrology, Children’s Hospital, Maternal and Child Health Hospital of Guangxi Zhuang Autonomous Region, Nanning, China

**Keywords:** acute respiratory infections, children, intensive care units, respiratory syncytial virus, risk factors

## Abstract

**Objectives:**

To analyze the clinical characteristics of hospitalized children with respiratory syncytial virus (RSV) infection and identify independent risk factors for intensive care unit (ICU) admission.

**Methods:**

This retrospective study included hospitalized children with polymerase chain reaction (PCR)-confirmed RSV infection between July 2022 and April 2025. Epidemiological, clinical, laboratory, and imaging data were collected. Multivariable logistic regression analysis was employed to determine independent predictors of ICU admission.

**Results:**

Of 95,311 children tested for acute respiratory infections, 14,583 (15.3%) were RSV-positive, of whom 5,814 (39.9%) were hospitalized. The hospitalized cohort was predominantly infants under one year of age (47.2%). The most common manifestations were cough (91.6%), sputum production (69.3%), and fever (55.8%). Laboratory findings revealed elevated median serum levels of procalcitonin, creatine kinase-MB, and lactate dehydrogenase. Chest computed tomography primarily revealed increased bronchial wall thickening (87.3%) and patchy shadows (67.2%). Complications were frequent, affecting the respiratory system in 53.4% of cases and other systems in 33.5%. A total of 710 children (12.2%) required ICU admission. To identify early warning predictors, a multivariable logistic regression model excluding acute clinical presentations (shortness of breath and dyspnea) identified respiratory complications (OR = 5.20, 95% CI: 3.82–7.07, *P* < 0.001), bilateral consolidation on chest imaging (OR = 2.08, 95% CI: 1.52–2.85, *P* < 0.001), and prolonged fever duration (OR = 1.19 per day, 95% CI: 1.14–1.23, *P* < 0.001) as the strongest independent risk factors for ICU admission.

**Conclusions:**

Hospitalized children with RSV infection are predominantly young infants and are prone to complex manifestations and multi-system complications. Respiratory complications, bilateral consolidation, and prolonged fever duration are key predictors of ICU admission. This study provides important evidence for the early identification of high-risk children and the optimization of critical care resource allocation.

## Introduction

Respiratory syncytial virus (RSV) is a leading cause of acute respiratory infection (ARI) in infants and young children, with the greatest burden attributable to acute lower respiratory tract infections (LRTIs) (Mazur et al., 2024). Globally, RSV accounts for an estimated 22% of all pediatric ARIs (Regassa et al., 2023). In 2019, approximately 33 million episodes of RSV-associated acute LRTI occurred worldwide in children under five years, resulting in 3.6 million hospitalizations and 26,300 in-hospital deaths. The burden in China is similarly substantial, with an estimated 3.5 million RSV-associated LRTI cases, 623,500 hospitalizations, and 2,650 in-hospital deaths among children under five in the same year (Li et al., 2022).

Although risk factors for RSV-related hospitalization—such as young age, prematurity, and chronic comorbidities—are well established, predictors of progression to critical illness (e.g., ICU admission or death) remain poorly characterized (Wang et al., 2024; Dallagiacoma et al., 2025). Existing studies are often constrained by narrow scopes, focusing on isolated epidemiological or clinical features without comprehensively integrating laboratory, imaging, and complication profiles from large hospitalized cohorts. Moreover, the relatively low incidence of severe endpoints has limited statistical power and hindered consensus on robust risk factors.

Recent advances in RSV immunoprophylaxis—including long-acting monoclonal antibodies and maternal vaccines—are increasingly being integrated into public health policies worldwide (Ares-Gómez et al., 2024; Barnett et al., 2025). The effective implementation of these strategies requires precise identification of children at highest risk for severe disease, underscoring the need for a thorough characterization of severe RSV phenotypes and validated risk-prediction models.

To address these gaps, this study systematically characterizes the epidemiological, laboratory, imaging, and clinical features of RSV infection in hospitalized children from the Guangxi region of southern China, and employs multivariable regression to identify independent risk factors for ICU admission. Our findings aim to support early identification of high-risk children, guide targeted interventions, and inform the optimization of prevention strategies and critical care resource allocation.

## Materials and methods

### Study population

**Figure 1:**
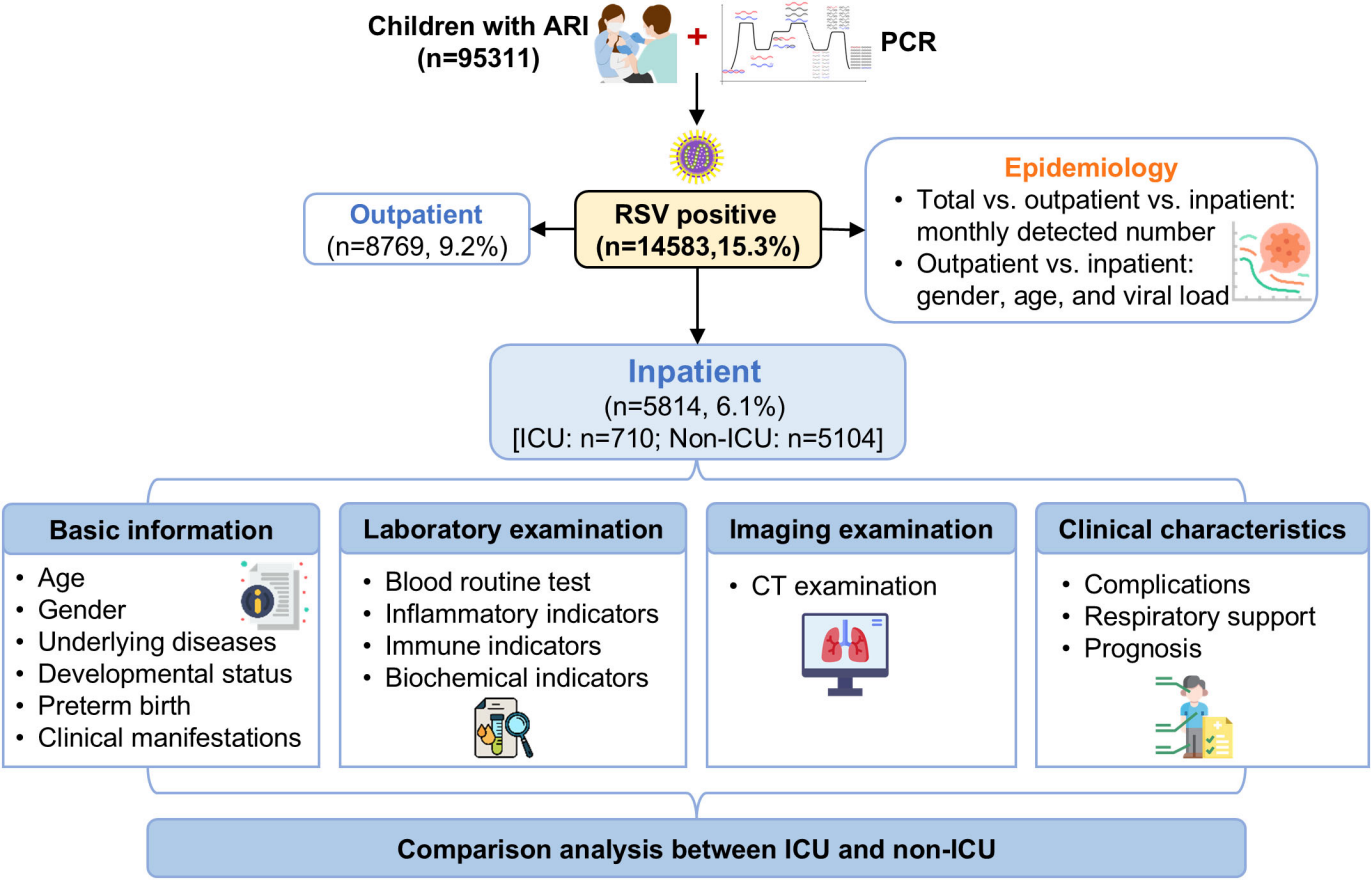
Flowchart of experimental design. ARI, acute respiratory infection; CT, computed tomography; ICU, intensive care unit.

This study retrospectively analyzed all children with acute respiratory infections (ARI) who presented at the Maternal and Child Health Hospital of Guangxi Zhuang Autonomous Region and underwent nucleic acid testing for RSV between July 2022 and April 2025. Based on the test results and clinical management, RSV-positive children were categorized into outpatient and inpatient groups (**Figure 1**). This study focused primarily on RSV-positive hospitalized children (the inpatient group), from whom clinical information and testing data were collected. The cohort horizon was defined as follows: the start of follow-up was the time of hospital admission, and the end of follow-up was hospital discharge or in-hospital death. ARI refers to an acute clinical illness characterized by the sudden onset of respiratory symptoms caused by respiratory pathogens (usually with a disease course not exceeding 21 days). The main symptoms of ARI include cough, sputum production, shortness of breath, sore throat, and runny nose. ARI mainly includes acute upper respiratory tract infections, acute bronchitis, and community-acquired pneumonia (CAP).

### Inclusion and exclusion criteria

Inclusion Criteria (all must be met): 1) Pediatric patients presenting with symptoms of acute respiratory infection; 2) Age ≤14 years; 3) Availability of essential key clinical or laboratory records (including demographic details, polymerase chain reaction (PCR) test reports, and confirmed clinical diagnoses).

Exclusion Criteria (excluded if any criterion applies): 1) Respiratory inflammation attributable to non-infectious causes (e.g., allergic reactions, asthma, or exposure to irritants); 2) Infections identified as hospital-acquired (nosocomial).

### Missing data and handling

All children who met the inclusion criteria during the study period (July 2022 to April 2025) were consecutively enrolled; no selective sampling was applied. Details of missing data and handling methods are provided in **Supplementary Table 1**. Specifically, for variables with a missing proportion of less than 5%, median imputation was applied for non-normally distributed data. For variables with a missing proportion between 5% and 20%, multiple imputation (m=5) was used. Variables with a missing proportion exceeding 20% (erythrocyte sedimentation rate and D-dimer) were excluded from further analysis.

### RSV detection method

Pharyngeal swab specimens were collected from pediatric patients. Nucleic acid extraction was performed using the Nucleic Acid Extraction Reagent (Shuoshi Biotechnology Co., Ltd., China; cat. SDKF60101) and real-time PCR amplification was conducted with a commercial kit (DAAN Gene Co., Ltd., Guangzhou, China; cat. DA0203) according to the manufacturer’s protocols. Real-time quantitative PCR was carried out on the SLAN-96P fully automated PCR analysis system (Hongshi Medical Technology, China) using a TaqMan probe targeting the N gene of human respiratory syncytial virus (HRSV; Gene ID: 1494470). The assay’s limit of detection (LOD) is 1000 copies/mL, and it has passed an external quality assessment organized by the National Center for Clinical Laboratories, China.

A specimen was considered positive if an amplification curve was observed and the cycle threshold (Ct) value was <36; negative if no amplification curve or Ct >40; samples with Ct values between 36 and 40 were retested. If the repeat test yielded Ct <36 or remained in the gray zone, the sample was deemed positive; if Ct >40, negative. This assay detects HRSV only and does not identify coinfecting pathogens.

### Chest imaging acquisition and interpretation

Chest radiography (CXR) was performed as the first-line imaging modality for all hospitalized children with suspected lower respiratory tract infection. Chest computed tomography (CT) was not performed routinely but was reserved for specific clinical indications, including: (i) suspected complicated pneumonia (e.g., parapneumonic effusion, empyema, necrotizing pneumonia, or lung abscess) with equivocal or inadequate findings on CXR; (ii) clinical deterioration or persistent high fever despite 48–72 hours of appropriate antibiotic therapy; (iii) preoperative evaluation prior to bronchoscopy or surgical intervention; (iv) immunocompromised status; and (v) suspected underlying anatomical abnormalities or foreign body aspiration. All chest CT images were independently reviewed by two radiologists who were blinded to the patients’ clinical outcomes and other clinical information. In cases of disagreement, a third senior radiologist was consulted, and a consensus interpretation was reached through discussion.

### Statistical methods

All data analyses were performed using IBM SPSS Statistics (version 26.0). The normality of continuous variables was evaluated using the Kolmogorov-Smirnov test with Lilliefors correction (**Supplementary Table 2**, **3**). Since all continuous variables deviated significantly from a normal distribution (all *P* < 0.05), they were described as median with interquartile range (IQR). For comparisons between two independent groups (ICU vs. non-ICU), the Mann-Whitney U test was used. For comparisons involving more than two independent groups, the non-parametric Kruskal–Wallis test was applied, followed by Dunn’s *post hoc* test. Categorical variables were summarized as frequencies and percentages (%), and differences between groups were examined using the chi−square test. To identify independent factors associated with ICU admission, multivariable logistic regression was employed. A sensitivity analysis using modified Poisson regression with robust error variance was additionally performed to estimate risk ratios (RRs). Statistical significance was defined as a two−tailed *P* value < 0.05.

## Results

### Detection of respiratory syncytial virus

Between July 2022 and April 2025, a total of 95,311 children with respiratory infections underwent PCR testing for RSV. Among them, 14,583 cases tested positive, yielding an overall positive rate of 15.3%. Of the positive children, 8,769 were outpatients (accounting for 9.2% of all tested children), and 5,814 were inpatients (accounting for 6.1% of all tested children). Among the 5,814 hospitalized children, 2,619 (45.0%) were transferred from other hospitals, and the remaining 3,195 (55.0%) were directly admitted to our hospital. The testing workflow is shown in **Figure 1**.

**Figure 2:**
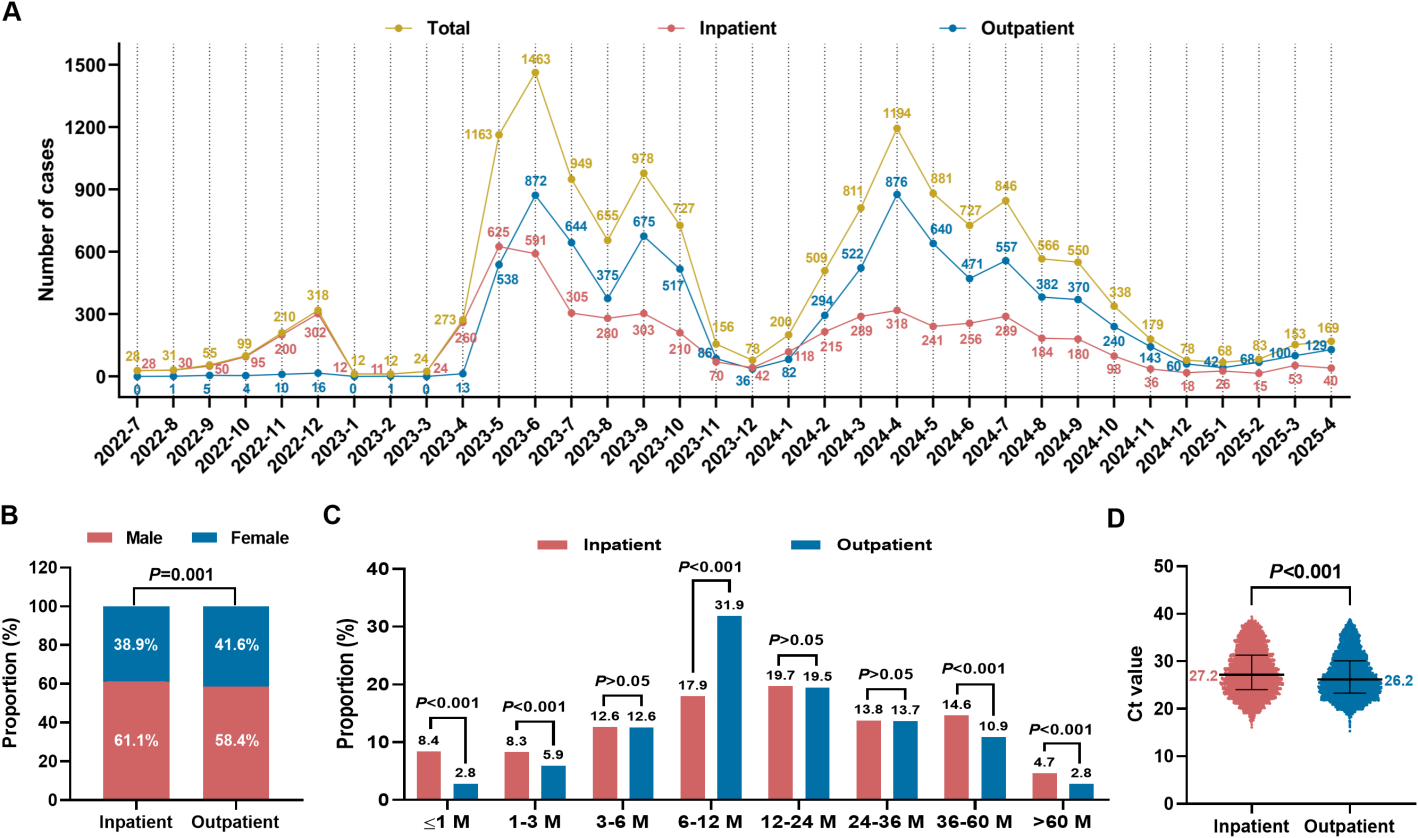
Epidemiological and clinical characteristics of children with RSV infection. **(A)** Monthly distribution of RSV cases. **(B)** Sex distribution comparison between inpatients and outpatients. **(C)** Age group distribution comparison between inpatients and outpatients. **(D)** Comparison of viral load (by PCR Ct value) between inpatients and outpatients.

Temporal distribution analysis revealed that RSV infection exhibited a distinct seasonal pattern, with the peak incidence occurring between April and July each year (**Figure 2A**). Compared with outpatient children, inpatient children showed significant differences in demographic characteristics: a higher proportion were male (**Figure 2B**), and they had a significantly higher distribution in the age groups of ≤1 month, 1–3 months, 36–60 months, and >60 months (*P* < 0.05) (**Figure 2C**). In addition, viral load (reflected inversely by PCR cycle threshold [Ct] values) was significantly higher in outpatients than in hospitalized children (*P* < 0.05) (**Figure 2D**).

### Baseline characteristics of hospitalized children with RSV infection

A total of 5,814 children hospitalized due to RSV infection were included in this study. The proportion of males was slightly higher (61.1%). Age distribution was skewed toward younger ages, with infants under 1 year constituting the largest group (47.2%), comprising children aged ≤1 month (8.4%), 1–3 months (8.3%), 3–6 months (12.6%), and 6–12 months (17.9%). Regarding underlying health conditions, hematologic diseases were the most common (498/5814, 8.6%), followed by respiratory system diseases (414/5814, 7.1%), and cardiovascular system diseases (343/5814, 5.9%). Most children (96.0%) had normal development, and 4.0% had developmental delay. Additionally, 11.4% had a history of preterm birth. Clinical manifestations were predominantly respiratory. Cough was the most common symptom (91.6%), followed by sputum production (69.3%), fever (55.8%), rhinorrhea (46.0%), and nasal congestion (38.0%). Symptoms suggesting lower respiratory tract involvement or greater severity, including wheezing (24.9%), shortness of breath (11.4%), and dyspnea (2.8%), were also documented (**Table 1**).

**Table 1:** Baseline characteristics of hospitalized children with RSV infection.

Baseline characteristics	Total population (N = 5814)	ICU (n=710)	Non-ICU (n=5104)	*P* value
Age (month), n (%)				
M ≤ 1	489 (8.4%)	140 (19.7%)	349 (0.8%)	***<0.001
1<M ≤ 3	482 (8.3%)	125 (17.6%)	357 (7.0%)	***<0.001
3<M ≤ 6	734 (12.6%)	115 (16.2%)	619 (12.1%)	**0.002
6<M ≤ 12	1043 (17.9%)	133 (18.7%)	910 (17.8%)	0.557
12<M ≤ 24	1144 (19.7%)	91 (12.8%)	1053 (20.6%)	***<0.001
24<M ≤ 36	802 (13.8%)	46 (6.5%)	756 (14.8%)	***<0.001
36<M ≤ 60	849 (14.6%)	37 (5.2%)	812 (15.9%)	***<0.001
M>60	271 (4.7%)	23 (3.2%)	248 (4.9%)	0.055
Gender, n (%)				
male	3553 (61.1%)	483 (68.0%)	3070 (60.1%)	***<0.001
female	2261 (38.9%)	227 (32.0%)	2034 (39.9%)	
Underlying diseases, n (%)				
Cardiovascular system diseases	343 (5.9%)	135 (19.0%)	208 (4.1%)	***<0.001
Nervous system diseases	138 (2.4%)	47 (6.6%)	91 (1.8%)	***<0.001
Respiratory system diseases	414 (7.1%)	100 (14.1%)	314 (6.2%)	***<0.001
Hematologic diseases	498 (8.6%)	102 (14.4%)	396 (7.8%)	***<0.001
Immune system diseases	316 (5.4%)	26 (3.7%)	290 (5.7%)	**0.009
Digestive system diseases	160 (2.8%)	60 (8.5%)	100 (2.0%)	***<0.001
Other systemic diseases	321 (5.5%)	91 (12.8%)	230 (4.5%)	***<0.001
Developmental status, n (%)				
Delayed development	233 (4.0%)	87 (12.3%)	146 (2.9%)	***<0.001
Normal development	5581 (96.0%)	623 (87.7%)	4958 (97.1%)	
Premature birth, n (%)				
Yes	663 (11.4%)	178 (25.1%)	485 (9.5%)	***<0.001
No	5151 (88.6%)	532 (74.9%)	4619 (90.5%)	
Clinical manifestation, n (%)				
Cough	5324 (91.6%)	641 (90.3%)	4683 (91.8%)	0.187
Expectoration	4032 (69.3%)	432 (60.8%)	3600 (70.5%)	***<0.001
Fever	3243 (55.8%)	576 (81.1%)	2667 (52.3%)	***<0.001
Rhinorrhea	2677 (46.0%)	166 (23.4%)	2501 (49.0%)	***<0.001
Nasal congestion	2209 (38.0%)	159 (22.4%)	2050 (40.16%)	***<0.001
Wheezing	1447 (24.9%)	327 (46.1%)	1120 (21.9%)	***<0.001
Vomiting	896 (15.4%)	118 (16.6%)	778 (15.2%)	0.341
Shortness of breath	660 (11.4%)	345 (48.6%)	315 (6.2%)	***<0.001
Diarrhea	415 (7.1%)	59 (8.3%)	356 (7.0%)	0.195
Dyspnea	165 (2.8%)	107 (15.1%)	58 (1.1%)	***<0.001
Abdominal pain	46 (0.8%)	4 (0.6%)	42 (0.8%)	0.465

*P < 0.05, **P < 0.01, and ***P < 0.001 represent statistically significant differences.

The bold values indicate statistical significance (P < 0.05).

### Laboratory findings of hospitalized children with RSV infection

Laboratory test results from 5,814 hospitalized children with RSV infection were analyzed (**Table 2**). The median serum levels of procalcitonin (PCT; 0.09 ng/ml, IQR: 0.06–0.17), creatine kinase-MB isoenzyme (CK-MB; 26 U/L, IQR: 22–32), and lactate dehydrogenase (LDH; 329 U/L, IQR: 290–378) were above the upper reference limits (typically PCT<0.05 ng/ml, CK-MB < 25 U/L, LDH 109–245 U/L). In contrast, the median level of immunoglobulin A (IgA; 0.4 g/L, IQR: 0.2–0.8) was below the lower reference limit for the pediatric range (typically 0.7–3.3 g/L). The median values for other routine parameters, including complete blood count, liver function, and renal function indicators, all fell within their respective normal reference ranges.

**Table 2:** Laboratory parameters and disease severity markers in hospitalized children with RSV infection.

Clinical features	Total population (N = 5814)	ICU (n=710)	Non-ICU (n=5104)	*P* value
Quantitative data [M (25%,75%)]				
RBC (×10^12^/L)	4.6 (4.2, 4.9)	4.1 (3.6, 4.6)	4.6 (4.3, 4.9)	***<0.001
HGB (g/L)	117 (108, 125)	107 (96, 119)	118 (110, 126)	***<0.001
WBC (×10^9^/L)	9.2 (7.1, 12.1)	8.7 (6.5, 11.9)	9.3 (7.1, 12.1)	***<0.001
NEU (%)	39.4 (25.1, 55.6)	44.6 (27.6, 60.3)	38.8 (24.8, 54.8)	***<0.001
LYM (%)	47.9 (33.4, 62.1)	44.0 (30.0, 57.2)	48.5 (33.9, 62.5)	***<0.001
Mon (%)	8.9 (6.9, 11.4)	8.6 (6.4, 11.2)	9.0 (7.0, 11.4)	**0.006
Eos (%)	1.1 (0.3, 2.6)	0.8 (0.1, 2.6)	1.1 (0.3, 2.6)	***<0.001
Bas (%)	0.3 (0.1, 0.4)	0.3 (0.1, 0.4)	0.3 (0.1, 0.4)	0.848
PLT (×10^9^/L)	360 (285, 449)	381 (302, 472)	357 (283, 445)	***<0.001
CRP (mg/L)	2.7 (0.6, 8.1)	2.4 (0.6, 7.7)	2.8 (0.6, 8.2)	0.440
PCT (ng/mL)	0.09 (0.06, 0.17)	0.11 (0.09, 0.21)	0.09 (0.06, 0.16)	***<0.001
LDH (U/L)	329 (290, 377)	322 (278, 377)	330 (292, 377)	**0.003
CK (U/L)	94 (68, 133)	85 (57, 132)	94 (69, 134)	***<0.001
CK-MB (U/L)	26 (22, 32)	28 (22, 36)	26 (22, 32)	***<0.001
AST (U/L)	39 (32, 48)	38 (30, 50)	39 (33, 47)	0.122
ALT (U/L)	18 (13, 25)	20 (15, 32)	17 (13, 24)	***<0.001
URE (umol/L)	23 (19, 28)	20 (16, 24)	23 (19, 28)	***<0.001
CYs-C (mg/L)	0.97 (0.84, 1.15)	1.06 (0.87, 1.28)	0.96 (0.84, 1.13)	***<0.001
IgA (g/L)	0.4 (0.2, 0.8)	0.3 (0.1, 0.6)	0.4 (0.2, 0.8)	***<0.001
IgG (g/L)	7.2 (5.2, 9.1)	6.6 (4.5, 8.7)	7.2 (5.3, 9.2)	***<0.001
IgM (g/L)	1.0 (0.7, 1.4)	0.9 (0.6, 1.3)	1.1 (0.7, 1.4)	***<0.001
Length of hospitalization (days)	6 (4, 8)	13 (9, 21)	5 (4, 7)	***<0.001
Treatment expense (CNY)	5373 (4140, 8713)	33285 (17596, 51514)	5035 (4008, 7031)	***<0.001
Duration of fever (days)	1 (0, 2)	3 (1, 7)	1 (0, 2)	***<0.001

*P < 0.05, **P < 0.01, and ***P < 0.001 represent statistically significant differences. WBC, White blood cell; NEU, Neutrophil; LYM, Lymphocyte; Mon, Monocyte; Eos, Eosinophil; Baso, Basophil; RBC, Red blood cell; PLT, Platelet; CRP, C-reactive protein; PCT, Procalcitonin; SAA, Serum amyloid A; ESR, Erythrocyte sedimentation rate; LDH, Lactic acid dehydrogenase; CK, Creatine kinase; CK-MB, Creatine kinase-MB; AST, Aspartate aminotransferase; ALT, Alanine aminotransferase; URE, Urea; CYs-C, Cystatin C; CNY, Chinese Yuan.

The bold values indicate statistical significance (P < 0.05).

### Viral load analysis

**Figure 3:**
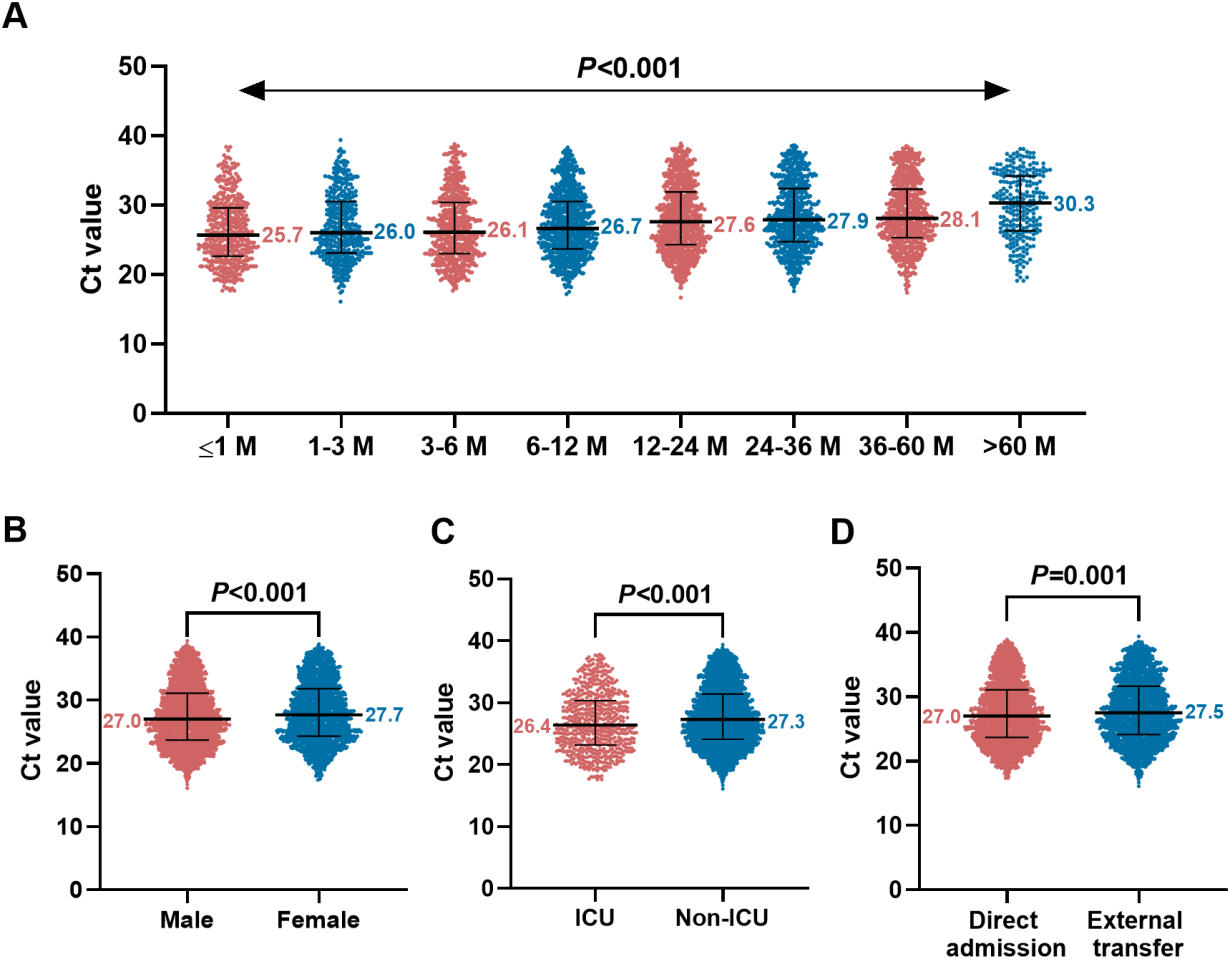
Analysis of respiratory syncytial virus (RSV) viral load in hospitalized children. **(A)** Distribution of PCR cycle threshold (Ct) values across different age groups of patients. **(B)** Comparison of Ct values between different genders. **(C)** Comparison of Ct values between patients admitted to the intensive care unit (ICU) and non-ICU patients. **(D)** Comparison of Ct values between children directly admitted to our hospital and those transferred from other hospitals.

To further explore the relationship between age and viral burden, we analyzed RSV viral load among hospitalized children of different ages. The results showed that median Ct values exhibited an increasing trend with age (**Figure 3A**). Since Ct value is inversely correlated with viral load, this trend indicates that viral load gradually decreases with age. Furthermore, viral load was significantly higher in male children than in female children (**Figure 3B**). Meanwhile, viral load was significantly higher in children admitted to the ICU than in non-ICU children (**Figure 3C**), and was also significantly higher in children directly admitted to our hospital than in those transferred from other hospitals (**Figure 3D**) (all *P* < 0.05).

### Chest computed tomography finding*s*

Among the 5,814 hospitalized children with RSV infection, 2,143 (36.9%) underwent chest CT. Imaging analysis revealed diverse pulmonary changes. The most common findings were prominent bronchial wall thickening (1,870 cases, 87.3%) and patchy opacities (1,440 cases, 67.2%). Consolidation was also frequent, observed bilaterally in 411 cases (19.2%) and unilaterally in 218 cases (10.2%). Other common manifestations included linear opacities (339 cases, 15.8%) and ground-glass opacities (219 cases, 10.2%). Less frequently, findings suggestive of small airway disease or complexity were observed, such as the tree-in-bud sign (67 cases, 3.1%), mosaic attenuation (35 cases, 1.6%), and atelectasis (61 cases, 2.8%). Complications such as pleural effusion (55 cases, 2.6%) and mediastinal lymph node enlargement (24 cases, 1.1%) were uncommon (**Table 3**).

**Table 3:** Chest imaging findings, complications, and clinical outcomes in hospitalized children with RSV infection.

Clinical features	Total population (N = 5814)	ICU (n=710)	Non-ICU (n=5104)	*P* value
Imaging characteristics	n^†^=2143	n^†^=404	n^†^=1739	
Bronchial wall thickening	1870 (87.3%)	351 (86.9%)	1519 (87.3%)	0.808
Ground-glass opacities	219 (10.2%)	42 (10.4%)	177 (10.2%)	0.916
Mosaic attenuation	35 (1.6%)	1 (0.2%)	34 (2.0%)	*0.015
Tree-in-bud sign	67 (3.1%)	1 (0.2%)	66 (3.8%)	***<0.001
Patchy opacities	1440 (67.2%)	298 (73.8%)	1142 (65.7%)	***<0.001
Linear opacities	339 (15.8%)	106 (26.2%)	233 (13.4%)	***<0.001
Reticular opacities	2 (0.1%)	1 (0.2%)	1 (0.1%)	0.394
Unilateral consolidation	218 (10.2%)	44 (10.9%)	174 (10.0%)	0.586
Bilateral consolidation	411 (19.2%)	173 (42.8%)	238 (13.7%)	***<0.001
Atelectasis	61 (2.8%)	36 (8.9%)	25 (1.4%)	***<0.001
Mucus plug sign	1 (0.1%)	0 (0.0%)	1 (0.1%)	1.000
Bronchiectasis	22 (1.0%)	10 (2.5%)	12 (0.7%)	***<0.001
Pleural effusion	55 (2.6%)	15 (3.7%)	40 (2.3%)	0.089
Pleural thickening	6 (0.3%)	1 (0.2%)	5 (0.3%)	1.000
Mediastinal lymph node enlargement	24 (1.1%)	2 (0.5%)	22 (1.3%)	0.170
Pulmonary embolism	2 (0.1%)	1 (0.2%)	1 (0.1%)	0.394
Necrotizing pneumonia	1 (0.1%)	0 (0.0%)	1 (0.1%)	1.000
Respiratory complications	1554 (26.7%)	535 (75.4%)	1019 (20.0%)	***<0.001
Other system complications	2080 (35.8%)	401 (56.5%)	1679 (32.9%)	***<0.001
Respiratory support	1319 (22.7%)	530 (74.6%)	789 (15.5%)	***<0.001
Adverse outcomes	125 (2.1%)	46 (6.5%)	79 (1.5%)	***<0.001

*P < 0.05, **P < 0.01, and ***P < 0.001 represent statistically significant differences. † Number of patients who actually underwent chest CT imaging.

The bold values indicate statistical significance (P < 0.05).

### Complications

Among all 5,814 hospitalized children, a total of 3,102 cases (53.4%) developed respiratory complications. The most common was sinusitis (957 cases), followed by hypoxemia (793 cases), pulmonary consolidation (629 cases), respiratory failure (541 cases), and adenoid hypertrophy (220 cases). Additionally, 1,947 cases (33.5%) experienced other system complications. The most frequent among these was gastrointestinal dysfunction (791 cases), followed by myocardial injury (246 cases), electrolyte disturbances (245 cases), trace element deficiencies (194 cases), and hepatic impairment (191 cases).

### Respiratory support

Among 5,814 hospitalized children with RSV infection, 1,319 (22.7%) received respiratory support. The majority of these children (921 cases) received non-invasive support, primarily via nasal cannula oxygen therapy (886 cases), with a smaller number using face mask oxygen therapy (12 cases) or continuous positive airway pressure (23 cases). Additionally, 398 children required invasive respiratory support (i.e., mechanical ventilation following endotracheal intubation or tracheostomy).

### Prognosis

The median length of hospital stay for children hospitalized with RSV infection was 6 days (IQR: 4–8 days), with a median hospital cost of 5,373 CNY (IQR: 4,140–8,713 CNY). A total of 710 children (12.2%) required ICU care due to a critical condition. The vast majority of children (5,689 cases, 97.9%) improved and were discharged after treatment. In 125 cases (2.1%), family members requested discharge against medical advice for personal reasons. No deaths occurred during hospitalization among all included children.

### Comparison between ICU and Non−ICU cases

Based on **Table 1**, baseline characteristics differed significantly between ICU and non−ICU children. The ICU group had a younger age distribution, with a markedly higher proportion of infants aged ≤3 months (37.3% [265/710] vs. 13.8% [706/5104]; *P* < 0.001). Proportions of males (68.0% vs. 60.1%), underlying conditions (e.g., cardiovascular disease: 19.0% vs. 4.1%; respiratory disease: 14.1% vs. 6.2%), developmental delay (12.3% vs. 2.9%), and preterm birth history (24.8% vs. 9.1%) were also significantly higher in the ICU group (all *P* < 0.001). Clinical manifestations such as fever (81.1% vs. 52.3%), wheezing (46.1% vs. 21.9%), tachypnea (48.6% vs. 6.2%), and dyspnea (15.1% vs. 1.1%) were more frequent in ICU children (all *P* < 0.001).

Analysis of laboratory indicators (**Table 2**) showed significantly higher levels of PCT (median 0.11 ng/ml, IQR: 0.09–0.21) and CK-MB (median 28 U/L, IQR: 22–36) in the ICU group compared to the non-ICU group (median PCT 0.09 ng/ml, median CK-MB 26 U/L; both *P* < 0.001). In contrast, IgA (median 0.3 g/L vs. 0.4 g/L) and LDH (median 322 U/L vs. 330 U/L) were significantly lower in the ICU group (*P* < 0.01). Notably, although some complete blood count parameters differed significantly between groups (*P* < 0.05), their median values remained within normal reference ranges.

In terms of imaging features, complications, and clinical outcomes (**Table 3**), the ICU group exhibited significantly higher rates of patchy opacities (73.8% vs. 65.7%), bilateral consolidation (42.8% vs. 13.7%), linear opacities (26.2% vs. 13.4%), and atelectasis (8.9% vs. 1.4%) (all *P* < 0.001). The risk of both respiratory complications (75.4% vs. 20.0%) and complications in other organ systems (56.5% vs. 32.9%) was also significantly higher in this group (*P* < 0.001). Consequently, the ICU group had substantially greater need for respiratory support (74.6% vs. 15.5%) and a higher incidence of adverse outcomes (6.5% vs. 1.5%) (*P* < 0.001).

To further investigate bacterial co-infection and clarify its potential role in explaining the elevated PCT levels observed in the ICU group, we analyzed microbiological culture results from the hospitalized cohort. In total, bacterial cultures were performed in 5,734 patients (98.7% of the total cohort), including 702 (12.2%) ICU children and 5,032 (87.8%) non-ICU children. As shown in **Supplementary Table 4**, the majority of patients in both groups had negative cultures for both bacteria and fungi (58.5% [411/702] vs. 72.2% [3,633/5,032]; *P* < 0.001). Bacteria-positive only cultures were more frequently identified in the ICU group than in the non-ICU group (33.3% [234/702] vs. 26.6% [1,340/5,032]; *P* < 0.001), while the proportions of fungi-positive only and dual bacteria/fungi-positive cultures were relatively low in both groups. Notably, across all culture-negative and culture-positive subgroups, PCT levels were consistently higher in ICU patients than in non-ICU patients, with the highest PCT levels observed in the dual-positive subgroup (ICU: median 0.22 ng/mL; non-ICU: 0.11 ng/mL).

### Logistic regression analysis

**Figure 4:**
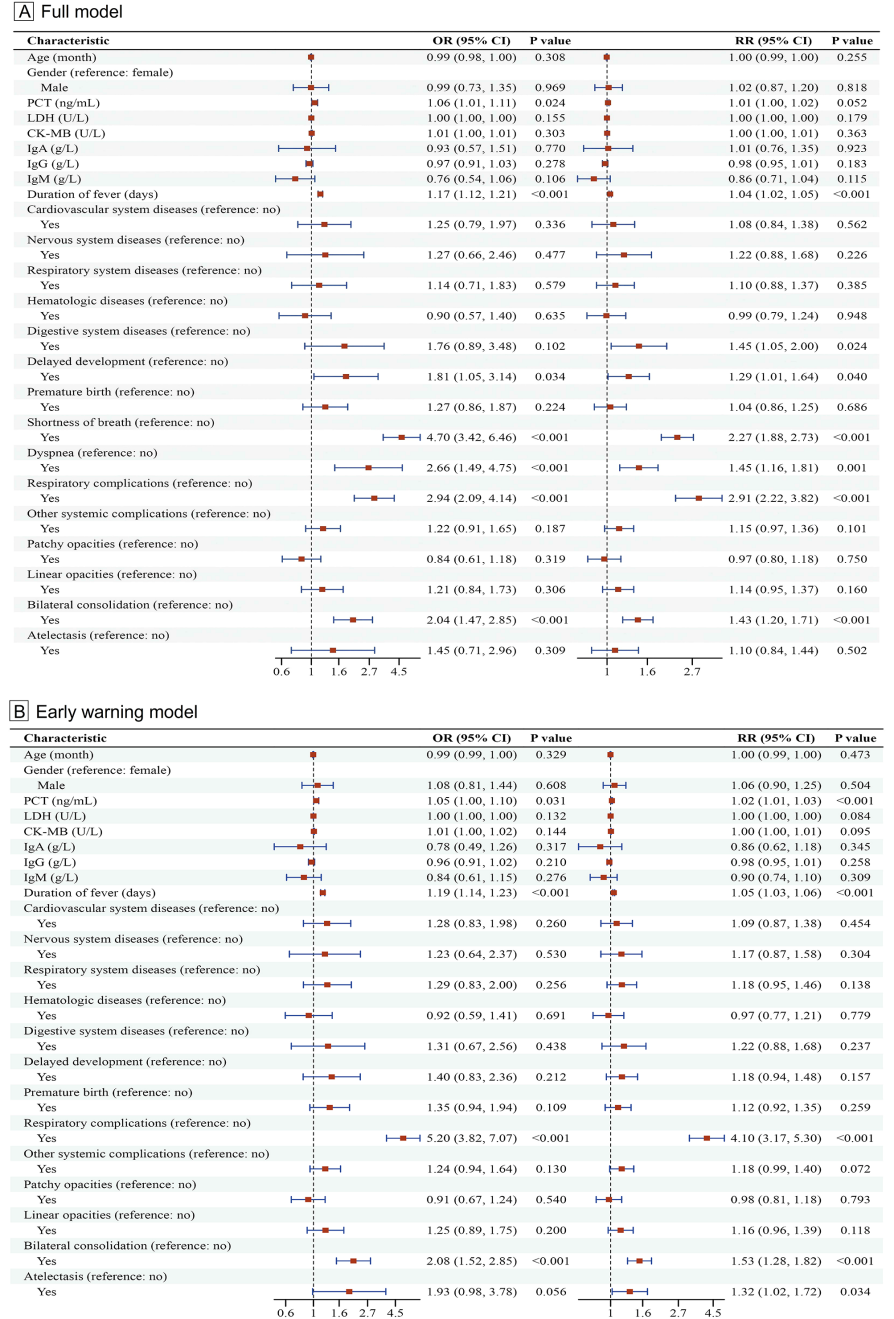
Multivariable logistic regression analysis of independent risk factors associated with ICU admission in children with RSV infection. **(A)** Multivariable model including all clinical, laboratory, and radiological variables. **(B)** Multivariable model excluding acute clinical symptoms (shortness of breath and dyspnea) to identify early warning predictors.

Multivariable logistic regression identified independent risk factors for ICU admission in children with RSV infection. Developmental delay was identified as an independent baseline risk factor (OR = 1.81, 95% CI: 1.05–3.14, *P* = 0.034). Clinical and laboratory markers independently associated with increased risk included prolonged fever duration (OR = 1.17 per day, 95% CI: 1.12–1.21, *P* < 0.001) and elevated PCT (OR = 1.06 per 1 ng/mL, 95% CI: 1.01–1.11, *P* = 0.024). Key indicators of disease severity were also significant, including shortness of breath (OR = 4.70, 95% CI: 3.42–6.46, *P* < 0.001), dyspnea (OR = 2.66, 95% CI: 1.49–4.75, *P* = 0.001), respiratory complications (OR = 2.94, 95% CI: 2.09–4.14, *P* < 0.001), and bilateral consolidation on chest imaging (OR = 2.04, 95% CI: 1.47–2.85, *P* < 0.001) (**Figure 4A**). Among these, the strongest independent predictor was shortness of breath (OR = 4.70).

To identify early “warning” predictors for ICU admission, we performed a secondary multivariable logistic regression analysis excluding acute clinical outcomes (shortness of breath and dyspnea). In this reduced model, prolonged fever duration (OR = 1.19 per day, 95% CI: 1.14–1.23, *P* < 0.001), elevated PCT (OR = 1.05 per 1 ng/mL, 95% CI: 1.00–1.10, *P* = 0.031), respiratory complications (OR = 5.20, 95% CI: 3.82–7.07, *P* < 0.001), and bilateral consolidation on chest imaging (OR = 2.08, 95% CI: 1.52–2.85, *P* < 0.001) remained independent risk factors for ICU admission. Atelectasis showed borderline significance (OR = 1.93, 95% CI: 0.98–3.78, *P* = 0.056) (**Figure 4B**). Respiratory complications emerged as the strongest predictor in this model (OR = 5.20).

We further applied modified Poisson regression to directly estimate risk ratios (RRs). The direction and significance of effects were largely consistent between OR and RR estimates (**Figures 4A, B**).

## Discussion

This study, conducted from July 2022 to April 2025, included 95,311 children with respiratory infections, yielding an overall RSV-positive rate of 15.3% (outpatient: 9.2%; inpatient: 6.1%). RSV infections showed a seasonal peak between April and July, consistent with epidemiological patterns in China (Xia et al., 2025; Wu et al., 2026). The COVID-19 policy changes dynamically influenced detection rates. During the containment period, comprehensive inpatient screening resulted in higher hospital detection rates. In contrast, after the policy was relaxed/post-COVID, the resurgence of outpatient visits led to outpatient RSV detection rates surpassing those in hospitals. Hospitalized children were predominantly male and exhibited a distinct age distribution, with higher proportions of infants ≤3 months—the highest-risk group due to immature immunity and airways—and children >36 months. This likely reflects selection bias, as many in the latter age group with mild symptoms recover at home without seeking care, leaving those with comorbidities or complex presentations overrepresented among hospital admissions. In contrast, infants aged 6–12 months constituted the largest outpatient group, possibly owing to partial prior immunity and greater physiological reserve, which facilitate outpatient management.

Further analysis of hospitalized children revealed a notably young age distribution, with infants under one year accounting for 47.2% of cases—a finding consistent with previous reports (Svensson et al., 2015; Glatman-Freedman et al., 2025). The observation that viral load (reflected inversely by Ct values) decreased with advancing age supports the hypothesis of improved viral clearance in older children. Underlying comorbidities, including hematologic, immune, or cardiovascular disorders, were also identified as significant risk factors for hospitalization, thereby amplifying the clinical burden of RSV infection. Clinically, although cough, sputum production, and fever were predominant, a considerable proportion of children presented with lower respiratory tract signs such as wheezing, tachypnea, and dyspnea, warranting close monitoring.

The laboratory and imaging findings provided important insights into the disease mechanisms and severity of RSV infection. In this study, a subset of hospitalized children exhibited elevated levels of procalcitonin, creatine kinase-MB isoenzyme, and lactate dehydrogenase, suggesting that RSV infection can induce a systemic inflammatory response and may involve the myocardium, leading to certain degrees of cardiopulmonary injury. Conversely, low serum immunoglobulin A (IgA) levels reflected the immaturity of mucosal immune function in young children, which may partly explain the immunological basis for their heightened susceptibility to RSV. Regarding imaging, chest CT commonly revealed bronchial wall thickening, patchy opacities, and consolidation—findings consistent with the imaging characteristics of RSV-associated bronchiolitis and pneumonia (Kern et al., 2001; Goolam et al., 2025)—thus providing an objective basis for clinical assessment.

This study further highlighted significant multisystem involvement as a key feature of RSV infection. Among hospitalized children, over half (53.4%) developed respiratory complications, with the most common being sinusitis (16.5%), hypoxemia (13.6%), and pulmonary consolidation (10.8%). Notably, the incidence of respiratory failure reached 9.3% (541 cases), indicating rapid disease progression in some children and underscoring the need for a high degree of clinical vigilance. Moreover, 33.5% of the children experienced extra-respiratory complications, predominantly gastrointestinal dysfunction (13.6%), myocardial injury (4.2%), and electrolyte disturbances (4.2%). These findings emphasize that clinical management of RSV should extend beyond the respiratory tract to include multi−organ monitoring, with particular attention to fluid balance and myocardial enzyme changes in infants and young children, to facilitate early identification and intervention of potential systemic effects.

The ICU admission rate in this study was 12.2%, which falls within the range of 1.8%–31% reported by global multicenter studies (Hedberg et al., 2024; Guarnieri et al., 2025; Lee et al., 2025). A retrospective study from three tertiary hospitals in South Korea involving 1,529 children reported an ICU admission rate of 1.8% (Lee et al., 2025). In a Swedish multicenter emergency department cohort study, the ICU admission rate for children with RSV infection was 2.9% (Hedberg et al., 2024). Conversely, a large-scale cohort study in the United States reported that approximately 26.8%–30.1% of infants hospitalized with RSV required ICU care (Law et al., 2025). These substantial variations likely reflect differences in enrollment periods, age distributions of study populations, and regional healthcare resource allocation. Notably, data from the Meyer Children’s Hospital in Florence, Italy (2019–2023), showed an ICU admission rate as high as 31% during the 2022–2023 season (Guarnieri et al., 2025). This increase has been attributed to post-pandemic “immunity debt” and the co-circulation of multiple respiratory viruses (Billard and Bont, 2023; Guarnieri et al., 2025). The 12.2% ICU rate observed in our study lies between the aforementioned figures. This finding reflects both the actual clinical profile of moderately to severely ill children admitted to our institution—a regional maternal and child health center—and aligns with the international trend toward increasing severity of RSV disease in the post-pandemic era.

Previous studies have established that key risk factors for severe RSV infection requiring ICU admission include young age (particularly <6 months), prematurity, chronic lung disease, winter birth, multiple birth, and underlying comorbidities (Li et al., 2022; Kirolos et al., 2025). Using multivariable logistic regression, the present study identified several independent risk factors significantly associated with ICU admission in children with RSV infection, with the strongest predictors being indicators of advanced disease severity: shortness of breath (OR = 4.70), dyspnea (OR = 2.66), respiratory complications (OR = 2.94), and bilateral consolidation (OR = 2.04), in addition to developmental delay (OR = 1.81), prolonged fever duration (OR = 1.17 per day), and elevated procalcitonin (OR = 1.06 per 1 ng/mL). Furthermore, to identify early “warning” predictors that precede overt respiratory failure, we performed a secondary multivariable logistic regression analysis excluding acute clinical outcomes (shortness of breath and dyspnea). In this reduced model, prolonged fever duration (OR = 1.19 per day), elevated PCT (OR = 1.05 per 1 ng/mL), respiratory complications (OR = 5.20), and bilateral consolidation (OR = 2.08) remained independent risk factors. These findings are clinically meaningful, as they support a three−tiered clinical risk pathway progressing from baseline vulnerability (e.g., developmental delay), to early inflammation and tissue injury (prolonged fever, elevated PCT, bilateral consolidation, respiratory complications), and ultimately to respiratory failure (tachypnea, dyspnea). The secondary analysis further underscores that factors from the early inflammatory phase can serve as critical warning signs, highlighting the value of dynamic monitoring of PCT and pulmonary imaging for early deterioration warning, timely intervention, and optimized allocation of critical care resources. Notably, PCT levels should be interpreted with caution, as they may be influenced by bacterial co−infection, antibiotic use, immune status, and disease duration. Currently, no RSV vaccine has been approved in China; however, the long−acting monoclonal antibody nirsevimab was approved in late 2023 and is being piloted in several provinces for high−risk infants (Guo et al., 2024; Li et al., 2024; Wang et al., 2025). These evolving strategies underscore the urgency of refining risk-stratification tools—such as the pathway proposed here—to guide targeted use of limited immunoprophylaxis resources and maximize public health impact.

Several limitations of this study should be acknowledged. First, as a single-center retrospective study, its generalizability may be limited. Chest CT was selectively used in children with suspected severe or complicated disease, which may have introduced selection bias and overestimated the association between radiological findings and ICU admission. Additionally, dynamic changes in COVID-19 policies during the study period may have influenced healthcare-seeking behaviors and testing strategies, potentially biasing the cohort composition. Second, pathogen detection relied on conventional targeted methods without broad-spectrum techniques (e.g., multiplex PCR), limiting our ability to comprehensively assess the impact of co-infections. Third, the study did not capture key therapeutic intervention details, such as time from symptom onset to medical care or from diagnosis to initiation of respiratory support. Fourth, some variables had missing data. Although the missing mechanism was likely missing completely at random due to documentation or data-recording errors rather than patient characteristics, this missingness introduces uncertainty in the data collection process, including whether some cases actually received the treatment. Therefore, the potential impact of this missingness should be acknowledged as a limitation.

In summary, this study identified several independent risk factors for ICU admission in children with RSV infection. Of these, shortness of breath, dyspnea, prolonged fever duration, elevated PCT, respiratory complications, and bilateral consolidation were significant predictors. Notably, the latter four also served as early warning predictors preceding overt respiratory failure. These findings support the implementation of an early warning and risk stratification system for at-risk children, which could facilitate timely intervention and optimize the allocation of critical care resources.

## Data Availability

The raw data supporting the conclusions of this article will be made available by the authors, without undue reservation.
