# Supplementary material for: Risk factors for ICU admission in hospitalized children with respiratory syncytial virus infection
Source: Front Cell Infect Microbiol. 2026 May 21;16:1834056. doi: 10.3389/fcimb.2026.1834056 (PMC13233450; doi:10.3389/fcimb.2026.1834056)
Supplement: Supplementary file 1 [file Table1.doc]

Supplementary Table 1. Missing data and handling methods for variables included in the analysis

| **Variable** | **Missing (n)** | **Missing(%)** | **Handling Method** |
| --- | --- | --- | --- |
| Age | 0 | 0 | — |
| Red blood cell | 4 | 0.07 | Median imputation |
| Hemoglobin | 4 | 0.07 | Median imputation |
| White blood cell | 4 | 0.07 | Median imputation |
| Neutrophil percentage | 5 | 0.09 | Median imputation |
| Lymphocyte percentage | 5 | 0.09 | Median imputation |
| Monocyte percentage | 5 | 0.09 | Median imputation |
| Eosinophil percentage | 5 | 0.09 | Median imputation |
| Basophil percentage | 5 | 0.09 | Median imputation |
| Platelet (PLT) | 4 | 0.07 | Median imputation |
| C-reactive protein (CRP) | 4 | 0.07 | Median imputation |
| Procalcitonin (PCT) | 234 | 4.02 | Median imputation |
| Erythrocyte sedimentation rate (ESR) | 1,170 | 20.12 | Excluded |
| D-dimer | 1,551 | 26.68 | Excluded |
| Lactate dehydrogenase (LDH) | 33 | 0.57 | Median imputation |
| Creatine kinase (CK) | 27 | 0.46 | Median imputation |
| CK-MB | 27 | 0.46 | Median imputation |
| Aspartate aminotransferase (AST) | 21 | 0.36 | Median imputation |
| Alanine aminotransferase (ALT) | 25 | 0.43 | Median imputation |
| Urea (URE) | 30 | 0.52 | Median imputation |
| Cystatin C (Cys-C) | 65 | 1.12 | Median imputation |
| Immunoglobulin A (IgA) | 941 | 16.19 | Multiple imputation (m=5) |
| Immunoglobulin G (IgG) | 942 | 16.20 | Multiple imputation (m=5) |
| Immunoglobulin M (IgM) | 942 | 16.20 | Multiple imputation (m=5) |
