# Supplementary material for: Risk factors for ICU admission in hospitalized children with respiratory syncytial virus infection
Source: Front Cell Infect Microbiol. 2026 May 21;16:1834056. doi: 10.3389/fcimb.2026.1834056 (PMC13233450; doi:10.3389/fcimb.2026.1834056)
Supplement: Supplementary file 2 [file Table2.docx]

Supplementary table 2. Normality test results for all participants (N = 5814)

| **Variable** | **Statistic** | **df** | **Sig.** |
| --- | --- | --- | --- |
| RBC (×10^12^/L) | 0.07 | 5814 | <0.001 |
| HGB (g/L) | 0.05 | 5814 | <0.001 |
| WBC (×10^9^/L) | 0.09 | 5814 | <0.001 |
| NEU (%) | 0.06 | 5814 | <0.001 |
| LYM (%) | 0.04 | 5814 | <0.001 |
| Mon (%) | 0.07 | 5814 | <0.001 |
| Eos (%) | 0.20 | 5814 | <0.001 |
| Bas (%) | 0.18 | 5814 | <0.001 |
| PLT (×10^9^/L) | 0.06 | 5814 | <0.001 |
| CRP (mg/L) | 0.33 | 5814 | <0.001 |
| PCT (ng/mL) | 0.43 | 5814 | <0.001 |
| LDH (U/L) | 0.10 | 5814 | <0.001 |
| CK (U/L) | 0.31 | 5814 | <0.001 |
| CK-MB (U/L) | 0.19 | 5814 | <0.001 |
| AST (U/L) | 0.25 | 5814 | <0.001 |
| ALT (U/L) | 0.30 | 5814 | <0.001 |
| URE (umol/L) | 0.21 | 5814 | <0.001 |
| CYs-C (mg/L) | 0.11 | 5814 | <0.001 |
| IgA (g/L) | 0.12 | 5814 | <0.001 |
| IgG (g/L) | 0.02 | 5814 | <0.001 |
| IgM (g/L) | 0.05 | 5814 | <0.001 |
| Length of hospitalization (days) | 0.28 | 5814 | <0.001 |
| Treatment expense (CNY) | 0.33 | 5814 | <0.001 |
| Duration of fever (days) | 0.29 | 5814 | <0.001 |

Kolmogorov-Smirnova test was used for normal distribution, the test output often applies Lilliefors correction to adjust the p-value. *P*<0.05 represents a non-normal distribution. WBC: White blood cell; NEU: Neutrophil; LYM: Lymphocyte; Mon: Monocyte; Eos: Eosinophil; Baso: Basophil; RBC: Red blood cell; PLT: Platelet; CRP: C-reactive protein; PCT: Procalcitonin; LDH: Lactic acid dehydrogenase; CK: Creatine kinase; CK-MB: Creatine kinase-MB; AST: Aspartate aminotransferase; ALT: Alanine aminotransferase; URE: Urea; CYs-C: Cystatin C; CNY: Chinese Yuan.
