# Supplementary material for: Risk factors for ICU admission in hospitalized children with respiratory syncytial virus infection
Source: Front Cell Infect Microbiol. 2026 May 21;16:1834056. doi: 10.3389/fcimb.2026.1834056 (PMC13233450; doi:10.3389/fcimb.2026.1834056)
Supplement: Supplementary file 3 [file Table3.docx]

Supplementary table 3. Normality test results for continuous variables by group (ICU vs. Non-ICU)

| **Variable** | **Group** | **Statistic** | **df** | **Sig.** |
| --- | --- | --- | --- | --- |
| RBC (×10^12^/L) | ICU | 0.05 | 710 | <0.001 |
|  | Non-ICU | 0.07 | 5104 | <0.001 |
| HGB (g/L) | ICU | 0.04 | 710 | 0.005 |
|  | Non-ICU | 0.05 | 5104 | <0.001 |
| WBC (×10^9^/L) | ICU | 0.11 | 710 | <0.001 |
|  | Non-ICU | 0.09 | 5104 | <0.001 |
| NEU (%) | ICU | 0.07 | 710 | <0.001 |
|  | Non-ICU | 0.06 | 5104 | <0.001 |
| LYM (%) | ICU | 0.04 | 710 | 0.009 |
|  | Non-ICU | 0.05 | 5104 | <0.001 |
| Mon (%) | ICU | 0.07 | 710 | <0.001 |
|  | Non-ICU | 0.08 | 5104 | <0.001 |
| Eos (%) | ICU | 0.24 | 710 | <0.001 |
|  | Non-ICU | 0.19 | 5104 | <0.001 |
| Bas (%) | ICU | 0.19 | 710 | <0.001 |
|  | Non-ICU | 0.17 | 5104 | <0.001 |
| PLT (×10^9^/L) | ICU | 0.07 | 710 | <0.001 |
|  | Non-ICU | 0.06 | 5104 | <0.001 |
| CRP (mg/L) | ICU | 0.33 | 710 | <0.001 |
|  | Non-ICU | 0.33 | 5104 | <0.001 |
| PCT (ng/mL) | ICU | 0.44 | 710 | <0.001 |
|  | Non-ICU | 0.41 | 5104 | <0.001 |
| LDH (U/L) | ICU | 0.18 | 710 | <0.001 |
|  | Non-ICU | 0.08 | 5104 | <0.001 |
| CK (U/L) | ICU | 0.21 | 710 | <0.001 |
|  | Non-ICU | 0.32 | 5104 | <0.001 |
| CK-MB (U/L) | ICU | 0.27 | 710 | <0.001 |
|  | Non-ICU | 0.15 | 5104 | <0.001 |
| AST (U/L) | ICU | 0.32 | 710 | <0.001 |
|  | Non-ICU | 0.20 | 5104 | <0.001 |
| ALT (U/L) | ICU | 0.35 | 710 | <0.001 |
|  | Non-ICU | 0.26 | 5104 | <0.001 |
| URE (umol/L) | ICU | 0.15 | 710 | <0.001 |
|  | Non-ICU | 0.23 | 5104 | <0.001 |
| CYs-C (mg/L) | ICU | 0.07 | 710 | <0.001 |
|  | Non-ICU | 0.12 | 5104 | <0.001 |
| IgA (g/L) | ICU | 0.15 | 710 | <0.001 |
|  | Non-ICU | 0.12 | 5104 | <0.001 |
| IgG (g/L) | ICU | 0.05 | 710 | <0.001 |
|  | Non-ICU | 0.03 | 5104 | <0.001 |
| IgM (g/L) | ICU | 0.07 | 710 | <0.001 |
|  | Non-ICU | 0.05 | 5104 | <0.001 |
| Length of hospitalization (days) | ICU | 0.22 | 710 | <0.001 |
|  | Non-ICU | 0.25 | 5104 | <0.001 |
| Treatment expense (CNY) | ICU | 0.20 | 710 | <0.001 |
|  | Non-ICU | 0.33 | 5104 | <0.001 |
| Duration of fever (days) | ICU | 0.19 | 710 | <0.001 |
|  | Non-ICU | 0.27 | 5104 | <0.001 |

Kolmogorov-Smirnova test was used for normal distribution, the test output often applies Lilliefors correction to adjust the p-value. *P*<0.05 represents a non-normal distribution. WBC: White blood cell; NEU: Neutrophil; LYM: Lymphocyte; Mon: Monocyte; Eos: Eosinophil; Baso: Basophil; RBC: Red blood cell; PLT: Platelet; CRP: C-reactive protein; PCT: Procalcitonin; LDH: Lactic acid dehydrogenase; CK: Creatine kinase; CK-MB: Creatine kinase-MB; AST: Aspartate aminotransferase; ALT: Alanine aminotransferase; URE: Urea; CYs-C: Cystatin C; CNY: Chinese Yuan.
