# Supplementary material for: Risk factors for ICU admission in hospitalized children with respiratory syncytial virus infection
Source: Front Cell Infect Microbiol. 2026 May 21;16:1834056. doi: 10.3389/fcimb.2026.1834056 (PMC13233450; doi:10.3389/fcimb.2026.1834056)
Supplement: Supplementary file 4 [file Table4.doc]

Supplementary Table 4. Procalcitonin (PCT) levels according to microbiology culture results among RSV-positive patients (n=5734)

| **Group** | **Subgroup** | **PCT (ng/mL)** | ***P* value** |
| --- | --- | --- | --- |
| Total patients with microbiology culture | ICU (n=702) | 0.11 (0.09, 0.21) | *****<0.001** |
|  | Non-ICU (n=5032) | 0.09 (0.06, 0.16) |  |
| Negative for both bacteria and fungi | ICU (n=411, 58.5%) | 0.11 (0.08, 0.17) | *****<0.001** |
|  | Non-ICU (n=3633, 72.2%) | 0.09 (0.06, 0.15) |  |
| Bacteria-positive only | ICU (n=234, 33.3%) | 0.12 (0.09, 0.31) | *****<0.001** |
|  | Non-ICU (n=1340, 26.6%) | 0.10 (0.07, 0.20) |  |
| Fungi-positive only | ICU (n=30, 4.3%) | 0.10 (0.09, 0.19) | 0.240 |
|  | Non-ICU (n=35, 0.7%) | 0.09 (0.05, 0.22) |  |
| Positive for both bacteria and fungi | ICU (n=27, 3.8%) | 0.22 (0.09, 0.98) | ***0.012** |
|  | Non-ICU (n=24, 0.5%) | 0.11 (0.07, 0.18) |  |

**P*<0.05, and ****P*<0.001 represent statistically significant differences. RSV: respiratory syncytial virus; ICU: intensive care unit; PCT: Procalcitonin.
